# Ultrasound Carotid Plaque Score and Severity of Coronary Artery Disease Assessed by Computed Tomography Angiography in Patients with Arterial Hypertension

**DOI:** 10.3390/diagnostics14111101

**Published:** 2024-05-25

**Authors:** Andrzej Wysocki, Michał Fułek, Piotr Macek, Monika Michałek-Zrąbkowska, Krzysztof Kraik, Małgorzata Poręba, Katarzyna Fułek, Helena Martynowicz, Grzegorz Mazur, Paweł Gać, Rafał Poręba

**Affiliations:** 1 Centre for Diagnostic Imaging, 4th Military Hospital, 50-981 Wroclaw, Poland; 2 Department of Internal Medicine, Occupational Diseases, Hypertension and Clinical Oncology, Wroclaw Medical University, 213 Borowska St., 50-556 Wroclaw, Poland; 3 Students’ Scientific Organization, Wroclaw Medical University, 50-556 Wroclaw, Poland; 4 Department of Paralympic Sports, Wroclaw University of Health and Sport Sciences, Witelona 25a, 51-617 Wroclaw, Poland; 5 Department and Clinic of Otolaryngology, Head and Neck Surgery, Wroclaw Medical University, 213 Borowska St., 50-556 Wroclaw, Poland; 6 Department of Population Health, Division of Environmental Health and Occupational Medicine, Wroclaw Medical University, Mikulicza-Radeckiego 7, 50-368 Wrocław, Poland

**Keywords:** carotid plaque score, coronary artery, arterial hypertension, coronary computed tomography angiography

## Abstract

The aim of the study was to assess the relationship between the presence of atherosclerotic lesions in the carotid arteries detected by ultrasound and the occurrence of atherosclerosis in the coronary arteries determined by computed tomography (CT) in patients with arterial hypertension (HTA). A total of 83 patients with HTA were qualified for the study (age: 71.3 ± 8.5 years). All subjects underwent carotid arteries ultrasound and coronary arteries CT. The carotid plaque score was assessed using ultrasound. The studied group was divided into two subgroups: a subgroup with the carotid plaque score ≤ 1 (A) and a subgroup with carotid plaque score ≥2 (B). Coronary arteries CT assessed coronary artery calcium score (CACS) and degree of coronary stenosis based on CAD-RADS. In subgroup B, a significantly higher CACS (411.3 ± 70.1 vs. 93.5 ± 31.8) and significantly higher grade in the CAD-RADS classification were demonstrated than in subgroup A (CAD-RADS ≥ 3: 21.8 vs. 6.0%). The regression analysis showed that carotid plaque score and age are independent risk factors for the severity of atherosclerotic lesions in the coronary arteries. In summary, ultrasound assessment of the carotid plaque score in patients with HTA could be considered as surrogate indicator of the risk and severity of atherosclerotic changes in the coronary arteries, but further studies are necessary to corroborate these results.

## 1. Introduction

Cardiovascular diseases (CVDs), primarily coronary artery disease (CAD) and stroke, are the predominant causes of death worldwide and a principal contributor to disability [1]. Hypertension remains one of the cornerstones of the cardiovascular risk (CVR) assessment [2].

Coronary angiography is of key importance in the imaging diagnosis of coronary artery disease. However, coronary angiography is an invasive method. In recent years, the importance of noninvasive imaging methods in the diagnosis of coronary artery disease has been increasing, primarily as complementary methods, and in selected groups of patients as optional methods compared to coronary angiography [3]. Cardiac computed tomography enables assessment of the risk of significant coronary disease (assessment of the coronary artery calcium score (CACS)) and assessment of the presence of atherosclerotic plaques in coronary arteries and the degree of coronary artery stenosis (computed tomography angiography), as well as assessment of reversible myocardial ischemia (perfusion with the administration of adenosine or regadenoson) [3,4]. Numerous studies are underway to develop the CCTA-derived fractional flow reserve (FFR-CT) technique [5]. Magnetic resonance imaging also enables the assessment of reversible myocardial ischemia in functional tests. Moreover, it allows the differentiation of ischemic myocardial injury from nonischemic causes, as well as the assessment of myocardial viability in patients with ischemic myocardial damage [3].

Ultrasonography is a simple, noninvasive technique that does not require radiation or intravenous contrast and is effective for risk prediction in atherosclerotic vascular disease [6]. The carotid arteries, being superficially situated and not obscured by bone, are particularly well suited for examination through ultrasonography. The ultrasound assessment of atherosclerotic plaques in the carotid arteries can be used to stratify cardiovascular risk in patients with arterial hypertension [7,8]. Taking into consideration the accessibility to carotid duplex ultrasound (CDUS) and its cost effectiveness [9], it seems important to search for the relationship between the presence and severity of atherosclerotic lesions in the carotid and coronary arteries.

## 2. Objectives

The aim of the study was to assess the relationship between the presence of atherosclerotic lesions in the carotid arteries detected by ultrasound and the occurrence of atherosclerosis in the coronary arteries determined with CCTA in patients with arterial hypertension.

## 3. Materials and Methods

### 3.1. General Characteristics of the Performed Study

The inclusion criteria for the study were age ≥ 18, arterial hypertension pharmacologically treated ≥5 years, and clinical indication to CCTA according to the recommendations of the European Society of Cardiology. The exclusion criteria were CACS value exceeded 800, insufficient quality of the coronary CT angiography, secondary hypertension, previous coronary interventions, previous myocardial infarction, previous stroke, chronic kidney disease, and hyperthyroidism or hypothyroidism.

Group size was determined using a sample size calculator. The selection conditions were as follows: population size 2.8 million (the size of the population from which patients were recruited for the study—the population size of the Lower Silesian Voivodeship in Poland), fraction size 0.3 (approximate prevalence of hypertension in the Polish population shown in population studies), maximum error 10% (standard level of maximum error used in scientific research), and confidence level 95% (standard level of statistical significance). The required minimum size of the study group was 81. We enrolled 83 patients with hypertension to the study: 37 (44.6%) men and 46 (55.4%) women; the average age of the subjects was 71.3 ± 8.5 years.

All subjects underwent ultrasound of the carotid arteries and CT of the coronary arteries. The studied group was divided into two subgroups: a subgroup with the carotid plaque score equal to or less than one and a subgroup with carotid plaque score equal to or greater than two.

### 3.2. Computed Tomography

The cardiac computed tomography (CCT) was performed using the standard coronary CT angiography (CCTA) protocol with dual-source 384-slice CT scanner SOMATOM Force (Siemens Healthcare, Erlangen, Germany). The obtained images were assessed by a certified radiologist with EACVI Cardiac Computed Tomography Exam and over 10 years of clinical experience. The coronary artery disease severity was determined based on the Coronary Artery Disease—Reporting and Data System (CAD-RADS), where 0—documented absence of coronary artery disease (CAD), 1—minimal nonobstructive CAD (maximal stenosis: 1–24%), 2—mild nonobstructive CAD (maximal stenosis: 25–49%), 3—moderate CAD (maximal stenosis: 50–69%), 4—severe CAD (maximal stenosis: 70–99%), and 5—total coronary artery occlusion.

### 3.3. Carotid Duplex Ultrasound

The ultrasound examination with carotid plaque score assessment was performed using a FUJIFILM Arietta 850 (FUJIFILM Healthcare Americas Corporation, Lexington, MA, USA) by the same experienced ultrasonographist in the case of all patients. The carotid plaque score was assessed in accordance with Recommendations for the Assessment of Carotid Arterial Plaque by Ultrasound for the Characterization of Atherosclerosis and Evaluation of Cardiovascular Risk: From the American Society of Echocardiography [10]. The carotid plaque score is a semiquantitative approach where the total number of carotid arteries segments containing plaque along the common carotid arteries, carotid bulbs, and internal carotid arteries are visualized and summed. Only the lesions seen in easily identified segments of the carotid arteries are considered for the assessment of the carotid plaque score. Therefore, lesions limited to the distal 1 cm of the common carotid arteries, the carotid bulb, and the proximal 1 cm of the internal carotid arteries were included in the evaluation. As a result, the assessment obtained carotid plaque scores ranging from 0 to 6.

### 3.4. Statistical Analysis

The statistical evaluations were conducted using “Dell Statistica 13” software (Dell Inc., Round Rock, TX, USA). Quantitative data were summarized as mean values ± standard deviations. The Shapiro–Wilk test was utilized to assess the distribution of the variables. Hypotheses testing for variables that followed a normal distribution was carried out using the t-test, whereas the Mann–Whitney U-test was applied for variables not normally distributed. Qualitative data were represented as percentages. For qualitative variables, the maximum likelihood chi-square test was used for further statistical analysis. To determine the relationship between the studied variables, correlation and regression analyses were performed. In the case of quantitative variables with a normal distribution, Pearson’s correlation coefficients were determined, in the case of quantitative variables with a non-normal distribution—Spearman’s coefficients, and in the case of ordinal variables—Kendall’s coefficients. The parameters of the model obtained in the regression analysis were estimated using the least squares method. Statistical significance was attributed to results with a *p*-value of less than 0.05.

## 4. Results

### 4.1. Clinical Characteristics in the Study Group

The detailed characteristics of the studied group is presented in Table 1.

Basing on the blood pressure readings that were obtained at the admission to the hospital, 37.3% of the participants were diagnosed with mild hypertension according to ESH/ECS. Moderate and severe grade of hypertension were assessed in 51.9% and 10.8% of the patients, respectively. The number of participants receiving combination therapy was 59 (71.1%), while monotherapy was found in 28.9% of participants. The average systolic blood pressure among the participants was 137.4 mmHg, with a standard deviation of ±16.2 mmHg, and the average diastolic blood pressure was 85.0 mmHg, with a standard deviation of ±7.9 mmHg.

### 4.2. Coronary Computed Tomography Angiography Parameters in the Study Group

There were various indications of CCTA among the patients included in the study. In 75.9% of patients, there was a suspicion of chronic coronary artery diseases, 48.2% of patients presented a chest pain, 30.1% had a low-intermediate CAD risk, 43.4% were at risk of numerous factors, 16.9% of patients had an inconclusive exercise test, and yielding nondiagnostic result of the exercise test was the reason for performing CCTA in a further 12% of participants. Four participants (4.8%) presented regional wall motion abnormalities of the left ventricle and one patient had a sudden cardiac death history in the family. The detailed summary is presented in Table 2.

Table 3 outlines the findings from the coronary computed tomography angiography (CCTA) in the study cohort. The participants had an average coronary artery calcium score of 249.3 (±67.3). The minimum CACS value was 0 and the maximum CACS value was 430.9. The median CACS in the study group was 245.3. In alignment with the coronary artery disease reporting and data system (CAD-RADS), which scores CAD presence from 0 (no CAD) to 5 (total coronary artery occlusion), 5 patients (6%) were scored as CAD-RADS 0, 29 patients (34.9%) scored 1, 39 (47%) scored 2, 6 (7.2%) fell into category 3, 3 (3.6%) were assessed as 4, and 1 (1.2%) was classified as 5.

### 4.3. Coronary Computed Tomography Angiography Parameters in the Study Subgroups Differing in a Carotid Plaque Score: A—Subgroup with the Carotid Plaque Score Equal to or Less Than One, B—Subgroup with Carotid Plaque Score Equal to or Greater Than Two

We evaluated the carotid plaque score, CACS and CAD-RADS across subgroups defined by the carotid plaque score. The subgroup with the carotid plaque score equal to or less than one was marked as subgroup A, and subgroup B stands for the subgroup with carotid plaque score equal to or greater than two. In general, the subgroup of patients with a carotid plaque score equal to or greater than two demonstrated a significantly higher coronary artery calcification index and more severe atherosclerotic changes in the coronary arteries based on the CAD-RADS in contrast to the subgroup of patients with a carotid plaque score equal to or less than one (*p* < 0.05). The profound results are summarized in Table 4.

### 4.4. Results of Correlation Analysis

Table 5 illustrates the outcomes from the correlation study, highlighting significant relationships between cardiovascular health metrics and various physiological measures. The research revealed notable correlations, underscoring the influence of age, body mass index (BMI), and total cholesterol on the scores of carotid plaque, coronary artery calcium score (CACS), and the Coronary Artery Disease—Reporting and Data System (CAD-RADS). Age consistently showed moderate correlations with all cardiovascular indicators. Similarly, BMI was moderately correlated with carotid plaque scores and CACS but did not correlate with CAD-RADS. High levels of total cholesterol were directly linked to increased carotid plaque scores and showed moderate associations with CACS and CAD-RADS. The study also identified specific relationships between systolic blood pressure and years of cigarette smoking with CACS, indicating their role in the development of coronary artery calcification. Conversely, diastolic blood pressure and fasting glucose levels, among others, did not show a significant correlation.

**Figure 1:**
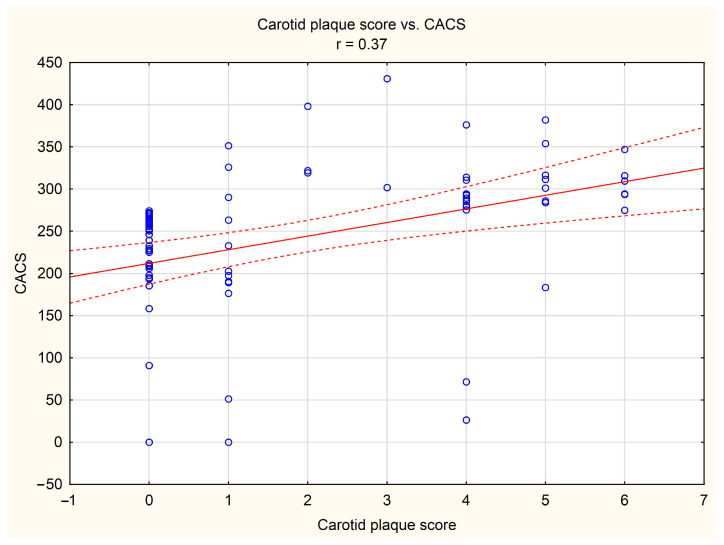
Correlation between carotid plaque score and coronary artery calcium score (CACS). Blue dots indicate individual cases, red line indicates the correlation line, and red dashed lines indicate the confidence interval (±95%) of the correlation line.

**Figure 2:**
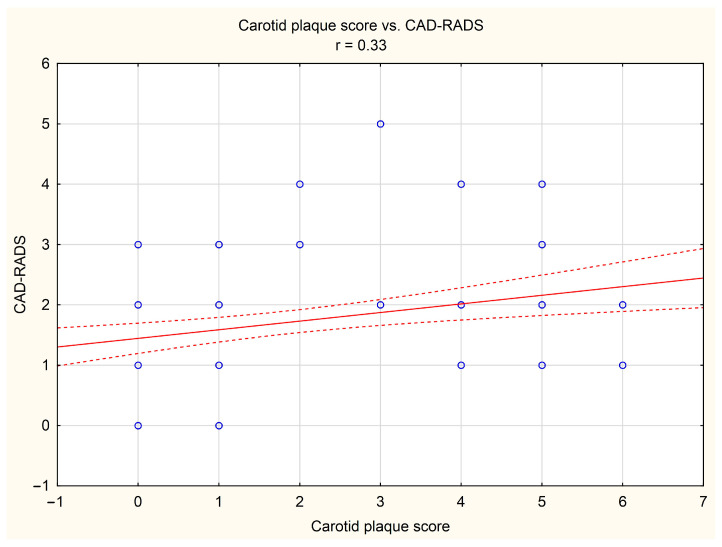
Correlation between carotid plaque score and Coronary Artery Disease—Reporting and Data System (CAD-RADS). Blue dots indicate individual cases, red line indicates the correlation line, and red dashed lines indicate the confidence interval (±95%) of the correlation line.

It was shown that in the study group, the carotid plaque score was positively correlated with tomographic indices of the risk and severity of coronary artery disease, i.e., with CACS (*r* = 0.37, *p* < 0.05, Figure 1) and CAD-RADS (*r* = 0.33, *p* < 0.05, Figure 2), respectively.

### 4.5. Results of Regression Analysis

In the study group, possible independent risk factors of tomographic indicators of risk and severity of coronary artery disease (CACS and CAD-RADS, respectively) were detected on the basis of univariable linear regressions between basic anthropometric data (age, BMI, gender), blood pressure values (systolic and diastolic), hypercholesterolemia and total cholesterol concentration in blood, type 2 diabetes and fasting blood glucose concentration, smoking and number of cigarette years, and carotid plaque score on the one hand, and variables from the CCTA examination on the other hand. In the next step, using multivariate stepwise regression analysis, considering statistically significant variables from univariate linear regressions, the final models were obtained separately for CACS (Table 6A) and CAD-RADS (Table 6B).

The obtained models indicate that older age and higher carotid plaque score values are independent risk factors for a higher risk of CAD, expressed by higher CACS values, and a higher CAD severity, expressed by a higher score in the CAD-RADS classification. Smoking is also associated with higher CACS values, regardless of other variables included in the analysis.

## 5. Discussion

In our study, it was observed that the subjects with a carotid plaque score equal to or greater than two had a significantly higher coronary artery calcification index and more severe atherosclerotic changes in the coronary arteries based on the CAD-RADS system in comparison to the subgroup of patients with a carotid plaque score equal to or less than one. Furthermore, we showed that the carotid plaque score and the patient’s age are independent risk factors for the severity of atherosclerotic lesions in the coronary arteries.

CCTA serves as a first-line imaging method to evaluate CAD in patients with stable chest pain [11]. It is capable of reliably excluding significant obstructive CAD with a high degree of luminal narrowing (≥50%) with high negative predictive value. It is widely acknowledged that this approach can indicate clinical treatments and reduce the incidence of myocardial infarction in comparison to conventional stress testing [12]. There are, however, some flaws. Firstly, CCTA is relatively expensive, and it demands the supply of contrast. Secondly, the performance of CCTA in patients with obstructive CAD is suboptimal due to its poor specificity. Severe calcific plaques forming luminal stenosis may be the reason for overestimation due to calcium blooming [13]. 

In a cohort of middle-aged individuals recruited from the general population, the carotid plaque score turned out to effectively predict the occurrence of stroke and major adverse cardiovascular events, surpassing the performance of SCORE2 in risk prediction [6]. According to the 2019 ESC Guidelines for the diagnosis and management of chronic coronary syndromes [14], however, carotid ultrasound intima media thickness (IMT) for cardiovascular risk assessment is not recommended in the general population [15]. Screening for CAD in asymptomatic subjects should base on a risk-estimation system such as SCORE2 scale. Total risk estimation is recommended for asymptomatic adults aged >40 years without evidence of CVD, diabetes, CKD, or familial hypercholesterolemia [14]. Recently, however, there has been performed a systematic review that supported the thesis that pictorial presentation of silent atherosclerosis using carotid US screening has a contributory role in CV risk stratification and prevention of CVD [16]. According to the meta-analysis performed by Gupta et al. [7], the presence of ultrasound-determined carotid plaque echolucency provides predictive information in asymptomatic carotid artery stenosis beyond luminal stenosis. Yet, the level of increased risk is inadequate by itself to pinpoint patients who would significantly benefit from surgical revascularization [7].

Carotid plaque, coronary artery calcium, or abnormal ankle pressures appear to be reliable markers for the existence of subclinical atherosclerosis and could be utilized as biomarkers [17]. The occurrence of cerebral ischemic incidents in patients with asymptomatic carotid stenosis is a significant concern. Plaque progression and contralateral stenosis are key factors in predicting these cerebral ischemic events [18]. Our results suggest that ultrasound assessment of the carotid plaque score in patients with arterial hypertension can be used as a prognostic indicator of the occurrence and severity of atherosclerotic changes in the coronary arteries. With plaque-RADS, the recently published standardized system of reporting carotid plaque composition and morphology via different imaging techniques, the efficient communications between both scientists and physicians of different specialties seems more reachable [19].

Our study contains several important limitations that should be mentioned. Firstly, the study was performed on a small group of patients. The group size is, indeed, sufficient, as confirmed by the sample size calculator, and the patients were properly qualified from the point of view of radiological protection for computed tomography (only 6% of patients with the final documented absence of coronary artery disease). However, for a more reliable analysis, a study on a larger group of patients would be justified. Secondly, subjective inclusion and exclusion criteria were adopted, e.g., at least 5 years of hypertension or CACS > 800 as a criterion for withdrawing from the angiographic phase of CCTA. It was possible to simply include additional patients with hypertension in the study and then include all variables potentially influencing the results in statistical analyses. However, the principles of radiological protection were followed when qualifying patients for CCTA. In terms of the study group, our group is also characterized by an overrepresentation of women and the older age. Moreover, the study included only a few patients with a high degree of coronary artery disease according to the CAD-RADS classification (CAD-RADS > 3 in only 12% of patients). In terms of research methodology, a significant limitation is the lack of data on the results of further diagnostics in patients classified as having CAD-RADS 3 based on CCTA. A limitation in the way the study is presented may be the fact that the indications for CCTA examination in Table 2 are presented based on the entries of doctors referring patients for CCTA examinations on referrals to the computed tomography laboratory. Furthermore, it should be remembered that this is an observational study and, hence, a snapshot of the characteristics of the study population. Based on such studies, no conclusions can be drawn regarding long-term clinical outcomes.

## 6. Conclusions

Ultrasound assessment of the carotid plaque score in patients with HTA could be considered as a surrogate indicator of the risk and severity of atherosclerotic changes in the coronary arteries, but further studies are necessary to corroborate these results.

## Figures and Tables

**Table 1:** Clinical characteristics and characteristics of hypotensive treatment in the study group.

Number	83
Age (years)	71.3 ± 8.5
BMI (kg/m^2^)	27.2 ± 2.9
Gender (%)	
Men	44.6
Women	55.4
Grades of arterial hypertension according to ESH/ECS (%)	
Mild	37.3
Moderate	51.9
Severe	10.8
Hypotensive treatment (%)	100.00
Monotherapy	28.9
Combination therapy	71.1
Hypotensive drugs (%)	100.00
ACE inhibitors	56.6
β-blockers	53.0
Diuretics	30.1
Calcium channel blockers	26.5
Angiotensin receptor blockers	7.2
Systolic blood pressure (mmHg)	137.4 ± 16.2
Diastolic blood pressure (mmHg)	85.0 ± 7.9
Type 2 of diabetes (%)	9.6
Fasting glucose (mg/dL)	117.4 ± 53.6
Hypercholesterolemia (%)	54.2
Total cholesterol (mg/dL)	219.3 ± 45.4
Smoking (%)	34.9
Cigarette years	267.6 ± 161.7

ACE—angiotensin-converting enzyme inhibitors; BMI—body mass index; ESC—European Society of Cardiology; ESH—European Society of Hypertension.

**Table 2:** Reasons to perform Coronary Computed Tomography Angiography among the patients.

Indication to Coronary Computed Tomography Angiography (%)	
Chronic CAD suspicion	75.9
Chest pain	48.2
Numerous CAD risk factors	43.4
Low intermediate CAD risk	30.1
Inconclusive exercise test	16.9
Nondiagnostic exercise test	12.0
Regional wall motion abnormalities of left ventricular	4.8
Sudden cardiac death in the family history	1.2

**Table 3:** Coronary computed tomography angiography parameters in the study group.

CACS	249.3 ± 67.3
CAD-RADS (%)	
0	6.0
1	34.9
2	47.0
3	7.2
4	3.6
5	1.2

CACS—coronary artery calcium score; CAD—coronary artery diseases; CCTA—coronary computed tomography angiography; RADS—reporting and data system.

**Table 4:** Coronary computed tomography angiography parameters in the study subgroups differing in a carotid plaque score: A—subgroup with the carotid plaque score equal to or less than one, B—subgroup with carotid plaque score equal to or greater than two.

	Subgroup A (*n* = 51)	Subgroup B ( *n*= 32)	*p*
Carotid plaque score	0.3 ± 0.2	4.4 ± 1.2	<0.05
CACS	93.5 ± 31.8	411.3 ± 70.1	<0.05
CAD-RADS (%)			
0	7.8	3.1	ns
1	43.1	21.9	<0.05
2	43.1	53.1	<0.05
3	2.0	15.6	<0.05
4	2.0	6.2	ns
5	2.0	0.0	ns
CAD-RADS ≥ 3 (%)	6.0	21.8	<0.05

CACS—coronary artery calcium score; CAD—coronary artery diseases; RADS—reporting and data system.

**Table 5:** Results of correlation analysis.

	Carotid Plaque Score	CACS	CAD-RADS
Age (years)	0.29	0.42	0.34
BMI (kg/m^2^)	0.32	0.44	ns
Systolic blood pressure (mmHg)	ns	0.31	ns
Diastolic blood pressure (mmHg)	ns	ns	ns
Fasting glucose (mg/dL)	ns	ns	ns
Total cholesterol (mg/dL)	0.49	0.47	0.30
Cigarette years	ns	0.46	ns
Carotid plaque score	1.00	0.37	0.33
CACS	0.37	1.00	0.41
CAD-RADS	0.33	0.41	1.00

Numerical values are statistically significant (*p* < 0.05) correlation coefficients (*r*) between studied variables; ns is absence of a statistically significant relationship in correlation analysis; BMI—body mass index; CACS—coronary artery calcium score; CAD—coronary artery diseases; RADS—reporting and data system.

**Table 6:** Results of regression analysis in the study group.

(**A**) Risk factors for higher coronary artery calcium score (CACS)				
Model for: CACS				
	Intercept	Age (years)	Smoking #	Carotid plaque score
Regression coefficient	50.496	4.962	181.634	112.055
SEM of Rc	13.485	1.742	71.348	36.177
*p*	<0.05	<0.05	<0.05	<0.05
(**B**) Risk factors for higher disease severity in the CAD-RADS classification				
Model for: CAD-RADS				
		Intercept	Age (years)	Carotid plaque score
Regression coefficient		1.311	0.051	1.317
SEM of Rc		0.378	0.015	0.549
*p*		<0.05	<0.05	<0.05

# Dichotomous variable, where 0—no, 1—yes; CACS—coronary artery calcium score; CAD—coronary artery diseases; RADS—reporting and data system; SEM—standard error of mean.

## Data Availability

The raw data supporting the conclusions of this article will be made available by the authors on request.
